# An Adaptive Heterogeneous Online Learning Ensemble Classifier for Nonstationary Environments

**DOI:** 10.1155/2021/6669706

**Published:** 2021-03-15

**Authors:** Tinofirei Museba, Fulufhelo Nelwamondo, Khmaies Ouahada

**Affiliations:** ^1^Applied Information Systems Department, University of Johannesburg, Johannesburg, South Africa; ^2^Department of Electrical and Electronic Engineering Sciences, University of Johannesburg, Johannesburg, South Africa

## Abstract

In recent years, the prevalence of technological advances has led to an enormous and ever-increasing amount of data that are now commonly available in a streaming fashion. In such nonstationary environments, the underlying process generating the data stream is characterized by an intrinsic nonstationary or evolving or drifting phenomenon known as concept drift. Given the increasingly common applications whose data generation mechanisms are susceptible to change, the need for effective and efficient algorithms for learning from and adapting to evolving or drifting environments can hardly be overstated. In dynamic environments associated with concept drift, learning models are frequently updated to adapt to changes in the underlying probability distribution of the data. A lot of work in the area of learning in nonstationary environments focuses on updating the learning predictive model to optimize recovery from concept drift and convergence to new concepts by adjusting parameters and discarding poorly performing models while little effort has been dedicated to investigate what type of learning model is suitable at any given time for different types of concept drift. In this paper, we investigate the impact of heterogeneous online ensemble learning based on online model selection for predictive modeling in dynamic environments. We propose a novel heterogeneous ensemble approach based on online dynamic ensemble selection that accurately interchanges between different types of base models in an ensemble to enhance its predictive performance in nonstationary environments. The approach is known as Heterogeneous Dynamic Ensemble Selection based on Accuracy and Diversity (HDES-AD) and makes use of models generated by different base learners to increase diversity to circumvent problems associated with existing dynamic ensemble classifiers that may experience loss of diversity due to the exclusion of base learners generated by different base algorithms. The algorithm is evaluated on artificial and real-world datasets with well-known online homogeneous online ensemble approaches such as DDD, AFWE, and OAUE. The results show that HDES-AD performed significantly better than the other three homogeneous online ensemble approaches in nonstationary environments.

## 1. Introduction

Ensembles of classifiers have been successfully used in a variety of applications including text classification and extraction such as keyword extraction in text classification [1], text classification based on supervised clustering [2], text genre classification based on language function analysis, and feature engineering [3]. Applications that generate data from nonstationary environments, where the underlying phenomenon changes over time, are becoming increasingly prevalent [4]. Examples include sensor networks, spam filtering systems, and intrusion detection systems. The prevalence of data stream applications makes the area of learning in nonstationary environments increasingly important, and one of the biggest challenges in data stream learning is to deal with concept drift; that is, the underlying concept may drift dynamically over time. The nonstationarity can be a result of, for example, seasonality or periodicity effects, changes in the user's habits or preferences, and hardware or software faults affecting a cyber-physical system. In such nonstationary environments, where the probabilistic properties of the data change over time, a nonadaptive model trained under the false stationarity assumption is bound to become obsolete in time and perform suboptimally at best or fail catastrophically at worst [4]. An avalanche of approaches based on homogeneous and heterogeneous ensembles to handle concept drift can be found in the literature and are focused on how to quickly detect or adapt to concept drift. An ensemble of classifiers for handling concept drift can be active or passive. Active ensemble approaches use drift detection methods to explicitly detect concept drift. If a drift is detected, new predictive models are typically created to learn the new concept, thus helping the system to recover from concept drift. Passive ensemble approaches do not use concept drift detection methods but maintain an ensemble of predictive models. Even though it is well known that various types of predictive models can provide a very different predictive performance depending on the problem being tackled, little work has been dedicated to the investigation of what type of predictive model is most adequate over time in nonstationary environments where each example is learned separately upon arrival and then discarded [5]. When delivering online learning, it is difficult to identify which type of machine learning algorithm is suitable to use as a base model due to the different amounts of data available to evaluate the base models. With the availability of more data, ensemble learning algorithms must be capable of identifying the type of base learners that work best for the application domain. A combination of different types of models creates diversity and often leads to better predictive performance.

Therefore, this paper proposes an adaptive online heterogeneous ensemble learning algorithm for nonstationary environments based on dynamic ensemble selection, known as Heterogeneous Dynamic Ensemble Selection based on Accuracy and Diversity (HDES-AD).

HDES-AD automatically selects the most representative models for a particular concept or emphasizes the selection of the most diverse and accurate base models to be used over an extended period of time in dynamic environments associated with concept drift. This enables the algorithm to store base models of different forms of diversity and accuracy and use them to optimize prediction performance to accurately adapt timeously to concept drift. HDES-AD is evaluated on artificially generated data streams and real-world data streams. The predictive performance of HDES-AD is compared with existing and representative homogeneous ensemble approaches such as DDD, OAUE, and the Active Fuzzy Weighting Ensemble (AFWE). Empirical experiments conducted indicate that HDES-AD performs significantly better than DDD, OAUE, and AFWE in the presence of concept drift in data streams. HDES-AD leverages the power of diversity by intelligently switching its base classifiers and exploiting its heterogeneity to maximize diversity. The use of more than one learning algorithm allows us to maximize diversity and control the diversity required for each concept. It also allows heterogeneity to be maintained for an extended period of time. In nonstationary environments, there is little time to perform any resampling of the data when training models, generally precluding the use of bagging, boosting, or related methods that resample training data [6].

This paper is further organized as follows.  Section 2 presents related work.  Section 3 provides a description of the proposed approach.  Section 4 outlines the experimental set-up and provides an empirical evaluation of the HDES-AD algorithm on homogeneous online ensembles.  Section 5 provides an analysis of the results and Section 6 sets out concluding remarks.

## 2. Related Work

Scenarios associated with concept drift are not uncommon, and a number of contemporary approaches have been proposed to address recurring concepts with minimum overheads. Many machine learning predictive models have emerged in the literature as candidate solutions and ensemble classifiers have demonstrated the ability to handle drifting concepts in nonstationary environments and Pratama et al. [7] provide good conceptual reviews. The focus is on online learning algorithms for handling concept drift. In terms of diversity, the ensembles are broadly classified into homogeneous and heterogeneous, taking into consideration the drift handling approaches, and the ensemble classifiers are further categorized into active and passive approaches. Most existing heterogeneous ensemble techniques rely on metalearning [8], and this helps in deciding which learning technique works well on what data. Xia et al. [9] proposed a novel heterogeneous ensemble credit model based on bstacking approach. The approach integrates the bagging algorithm with the stacking method. Chai et al. [10] proposed a heterogeneous ensemble consisting of a least-squares support vector machine and two radial basis function networks to enhance the reliability of ensembles of uncertainty estimators in surrogate-assisted evolutionary optimization of computationally expensive problems. However, the computation time for constructing heterogeneous ensembles may become excessively long when the number of training samples increases. A two-stage consumer risk modeling system that uses heterogeneous ensemble learning was proposed by Hajek and Papouskova [11]. The approach integrates class-imbalanced ensemble learning for predicting credit scoring. The two-stage ensemble is computationally expensive with prohibitive overheads. Nguyen et al. [12] included the fuzzy if-then rule-based metalearner in a heterogeneous ensemble system to capture the uncertainty in the outputs of the base classifiers. The algorithm was evaluated on thirty datasets and was shown to significantly outperform other algorithms that were homogeneous in nature. Idrees et al. [13] proposed the Heterogeneous Dynamic Weighted Majority (HDWM) ensemble that makes use of model learners of different types that are weighted to maintain ensemble diversity and includes a drift detection mechanism. The algorithm exhibited responsive adaptation, dealing appropriately with changing environments to increase the reliability and predictive accuracy of the algorithm. The algorithm assigns weights to classifiers and removes weak classifiers from the pool, compromising its ability to handle recurring concepts. The use of weights makes the algorithm slow in reflecting new concepts. The algorithm heavily depends on human predefined parameters. Most passive homogeneous ensemble learning approaches, that is, those that do not rely on a drift detection method, handle concept drift by maintaining an ensemble of base models and use weights to emphasize the models believed to best represent the current concept [10]. Among the passive homogeneous approaches is the Online Accuracy Updated Ensemble (OAUE) [14], which combines chunk-based and online ensemble methods. The prediction accuracy of the algorithm is heavily dependent on window size, and a small window size may lose track of sudden concept drift and a large window is susceptible to false concept detection. Dominant among the active homogeneous approaches is the Diversity for Dealing with Drifts (DDD) [15]. DDD is an online active ensemble learning approach that creates different ensembles with different levels of diversity to achieve robustness for different types of drifts. The approach uses one learning algorithm and uses a drift detection mechanism. The use of one base learner makes the algorithm to be devoid of much-needed diversity. Fan et al. [16] proposed a novel adaptive ensemble algorithm, the Active Fuzzy Weighting Ensemble (AFWE), to handle data streams involving concept drift. The algorithm uses a drift detection mechanism and assigns weights to instances. Experimental results on seven datasets indicate that the algorithm can shorten the recovery time of accuracy drop when concept drift occurs, adapt to different types of concept drift, and obtain better performance with less computational cost than the other adaptive ensembles. AFWE is devoid of diversity, and the use of weights makes it slow in converging to new concepts. The task of learning in nonstationary environments has also been tackled lately using deep learning. Among the work carried out using deep learning is the work of Ashfahani and Pratama [17] who proposed a deep learning continual learning algorithm called Autonomous Deep Learning (ADL). ADL uses a drift detection mechanism, and the Network Significance (NS) formula is used as a pruning strategy. The drift detection is likely to introduce false alarms. Models that are likely to handle recurring contexts may be pruned with the algorithm. Pratama et al. [7] presented a Neural Network with Dynamically Evolved Capacity (NADINE). Its network structure evolves automatically. NADINE uses soft forgetting and adaptive memory approaches to cater to catastrophic forgetting. Models that can be relevant in the future might be forgotten. The learning process with deep learning is slow, and the draw of concept drift is that for a high volume of nonstationary data streams where the actual drift is unknown in advance, the time it takes to predict may grow indefinitely [18]. To perform better than other supervised machine learning techniques, deep learning requires very large amounts of data. Complex data models make deep learning to be extremely expensive. To execute efficiently, deep learning requires expensive devices thereby increasing cost to the users.

### 2.1. Heterogeneous Dynamic Ensemble Selection with Accuracy and Diversity (HDES-AD)

The Heterogeneous Dynamic Ensemble Selection with Accuracy and Diversity (HDES-AD) maintains a dynamic pool of learners. Learners are selected based on accuracy and diversity using the dynamic ensemble selection criteria. The learners in the dynamic pool are tested in a prequential way on the current instance in the data stream to check if they are representative of the current concept. The same current instance is used to train the dynamic size pool of learners. In the event of a wrong global prediction or concept drift by the entire ensemble, the latest data instance is used to train a new classifier and that classifier is used to update the entire pool of learners, and learners that are diverse and representative of the current concept are selected. The size of the dynamic pool of learners is controlled by a predefined parameter. HDES-AD implements both active and passive approaches to handle concept drift, reduce the convergence time of new concepts, and efficiently handle different types of drifts. To implement a passive approach, HDES-AD removes learners with the least accuracy and diversity from the dynamic pool once their accuracy and diversity fall below a predefined measure. Both passive and active approaches restrict the ensemble size from growing indefinitely and thus reduce the computational costs and overheads while enabling the ensemble to remain heterogeneous. The active approach is implemented via drift detection, and when the global prediction of the ensemble is wrong, as indicated by a drift detection mechanism, HDES-AD resets the entire learning system. The predictions generated by the base learners are transferred into the drift detection mechanisms to detect concept drift and warnings. The learner with the least amount of accuracy and corresponding diversity is removed from the dynamic pool.

The HDES-AD is outlined in Algorithm 1. Each learner in the dynamic pool is assigned an accuracy and diversity measure. Each learner in the dynamic pool makes a prediction on an instance at each time step, where the instance is a vector representing attributes in a data stream. Accuracy and diversity values are reset and recalculated upon reset.

## 3. Drift Detection and Adaptation

Algorithm 2 provides an outline of active drift handling in HDES-AD. The learners in the dynamic pool are reset once drift is detected. Each learner in the dynamic pool is assigned the accuracy level and the amount of diversity to prevent the domination of previously learned models over the newly created models. When the warning state is detected, the learners in the dynamic pool are retrained and the accuracy and diversity numbers are recalculated. HDES-AD uses Yule's-Q Statistic [19] as a diversity measure to minimize the ensemble error. The diversity measure is recommended due to its simplicity and ease of interpretation.

HDES-AD uses the drift detection method (DDM) to detect drift. If concept drift is detected, the preserved models are adapted to fit the current data. DDM is an online learning system since it does not store the training instances for posterior use.

Algorithm 3 implements the passive drift handling mechanism in HDES-AD. In the event of a globally wrong prediction, a new learner is trained on the new data instance and added to the dynamic pool. The accuracy and diversity of new learners are computed.

## 4. Experimental Results and Analysis

This section investigates the efficiency of the HDES-AD in handling concept drift and compares its accuracy and drift handling capabilities with ensemble algorithms of a homogeneous type such as DDD, OAUE, and AFWE designed to handle concept drift. Friedman tests with their corresponding post hoc tests are performed to support the comparison of the algorithms on multiple data streams. The second set of experiments conducted concern the evaluation of computational resource usages such as CPU time and memory.

HDES-AD is developed in Java programming language using the Massive Online Analysis (MOA). All other algorithms are already included in the MOA framework which is used in an experimental environment. MOA is an open-source framework for learning data streams in evolving environments. The base learners used in HDES-AD are Multilayer Perceptrons and Hoeffding Trees with the idea of generating maximum diversity and controlling it.

### 4.1. Datasets

The artificial and real-world data streams used in the experiments are generated through the MOA workbench. We provide the characteristics of the artificial data streams for the MOA framework.

- Random Tree Generator (Recurring) generates a stream based on a randomly generated tree and builds a decision tree by randomly selecting attributes as split nodes and assigning random classes to them. The Random Tree Generator allows customizing the number of nominal and numeric attributes as well as the number of classes.
- The SEA Generator (sudden and gradual drift) is a synthetic data stream generator that aims to simulate concept drift over time. It generates random points in a three-dimensional feature space, but only the first two features are relevant.
- LED Generator (sudden drift) generates a stream defined by 7-segment LED display, and the task is to predict the digit 0–9. Concept drift is simulated by interchanging relevant attributes. Such a stream is generated by emulating a sudden drift by combining two distributions. We generate the first distribution with the LEDGenerator and the second distribution is generated using the LEDGeneratorDrift and one attribute comprises a drift.
- Waveform (sudden drift) Generator is a 3-class problem defined by 40 numerical attributes and shares its origin with the LED dataset. The problem is to predict one of the three waveform types.
- Covertype dataset. The dataset consists of the observations determined from the US Forest Service Region 2 Resource Information System (RIS) data. It contains 581,012 instances, 54 attributes, and no missing values. The task is to predict the type of forest cover based on cartographic variables such as elevation, slope, and soil type.
- The Spam e-mail dataset. It contains input attributes that represent a gradual concept drift from the SpamAssassin collection. The dataset consists of 500 attributes and two target classes, and the task is to predict whether an e-mail is spam or legitimate. The attributes represent the presence of a given word in the e-mail.
- KDDcup99. This dataset was used in the Third International Discovery and Data Mining Tools Competition. The competition task was to build a network intrusion detector, a predictive model capable of distinguishing between bad connections (intrusion or attack) and good (normal) connections. The KDD99 Cup dataset contains a standard set of data to be audited, which includes a wide variety of intrusions simulated in a military network environment. The dataset contains 42 attributes and 23 classes.
- Poker Hand dataset. The dataset consists of 1,000,000 instances and 11 attributes. Each record of the Poker Hand dataset is an example of a hand consisting of five playing cards drawn from a standard deck of 52. Each card is described using two attributes (suit and rank), with a total of 10 predictive attributes. There is one class attribute that describes the ‘pokerhand'.

### 4.2. Evaluation Configuration

This section investigates the behavior of our proposed adaptive heterogeneity ensemble classifier, HDES-AD in nonstationary environments associated with concept drift and compares its prediction accuracy, switching capabilities, and drift handling capability with the existing homogeneous ensemble-based approaches, namely, DDD [15], OAUE [5], and AFWE [16].

The prediction performance of our adaptive heterogeneity ensemble classifier and its ability to handle the concept is tested on artificial data streams and real-world datasets, and the corresponding ranks are determined and higher averages correspond to lower ranks. To validate the hypothesis, significance tests and post hoc comparison of ranks are carried out to determine the significance level and critical difference (CD). The predictive accuracies of HDES-AD, DDD, HEFT, and OAUE are presented in Table 1. The chi-square and *p* value are calculated according to the method described by Demsar [20]. At the level of significance of 0.05, the value of *p* indicates significant differences. The Nemenyi test is applied for pairwise comparison. It is evident from the prediction accuracy table that HDES-AD performs significantly better than the other 3 homogeneous ensembles in nonstationary time series data.

Table 1 shows the prediction accuracies and rankings of the four algorithms and the CPU time in seconds.

### 4.3. Evaluation of HDES-AD

The predictive capabilities of HDES-AD together with its model switching capabilities and drift handling capabilities are compared against existing and representative homogeneous ensembles such as DDD, OAUE, and AFWE tested on artificial and real-world datasets, and corresponding ranks are determined in such a way that higher averages represent lower ranks. Significant tests and post hoc comparisons on ranks are performed to determine significance level and critical differences. The predictive accuracies and CPU time of HDES-AD, DDD, OAUE, and AFWE are shown in Table 1.

As shown in Table 1, HDES-AD achieved the best accuracy in both synthetic and real-world datasets with all the three active and passive homogeneous ensembles. The ensemble size of the HDES-AD is dynamic; that is, they are growing and shrinking based on the predictive performance and the drift handling detection and capabilities. HDES-AD achieved higher accuracy on both synthetic and artificial datasets, and this can only be attributed to its heterogeneity. HDES-AD retains highly diverse classifiers, thus preserving previously learned concepts, and this helps HDES-AD to deal appropriately with recurring concepts. HDES-AD periodically includes new classifiers from the latest data chunks, and this helps it to deal with concept drift appropriately and to maintain and improve its predictive accuracy. DDD maintains a static ensemble and discards classifiers if the ensemble size reaches a predefined size, making it unable to handle recurring concepts.

Consistent performance trends across the two ensemble approaches can be observed. However, to draw meaningful conclusions, it is critical to determine if the performance differences are statistically significant. To accomplish this, we employ the standard methodology given by Demsar [20] to test for statistically significant performance differences among the four ensemble approaches over all datasets.

In this study, the nonparametric Friedman test [21] is firstly used to determine if there is a statistically significant difference between the rankings of the compared techniques. The Friedman test reveals how statistically significant differences are (*p* < 0.05) for each ensemble generation strategy. As recommended by Demsar [20], we perform the Nemenyi post hoc test on average rank diagrams. The best ranking algorithms are on the rightmost side of the diagram. The algorithms that do not differ significantly (*α* = 0.005) are connected with a line. From the CD plots, HDMES-AD outperforms the other homogeneous ensembles most of the time. Using the Friedman/Nemenyi approach with a cut-off of *α* = 0.05, the pairwise comparison between heterogeneous and homogeneous ensembles is provided.

**Figure 1:**
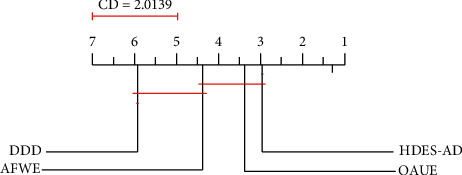
Average rank diagram on real-world datasets.

Figure 1 shows the critical difference plots from post hoc Nemenyi tests on all the datasets.

The nonparametric Friedman test was carried out to compare multiple classifiers over multiple datasets. Friedman's test was first used to determine if there is a statistically significant difference between the rankings of the compared techniques. The Nemenyi post hoc test on the average rank diagram was performed. The ranks are depicted on the axis in such a manner that the best ranking algorithms are at the rightmost side of the diagram. The algorithms that do not differ significantly (at *p*=0.05) are connected with a line. The critical difference (CD) is indicated above the graph. As can be observed from the CD plot, HDES is ranked first. However, its performances are not statistically distinguishable from the performances of OAUE, AFWE, and DDD according to the post hoc test despite the fact that the nonparametric statistical tests that were used are very conservative.

### 4.4. Kappa Evaluation Measures

Apart from the accuracy measure, MOA also provides the Kappa measure. The Kappa evaluation measure is widely used for learning data streams in evolving environments and has the ability to handle both multiclass and imbalanced class problems. A larger value of the Kappa evaluation measure is an indication of a more generalized classifier. Negative Kappa values are a sign of low prediction accuracy. Kappa values for both artificial and real-world datasets were positive for the heterogeneity ensemble and the other three homogeneous ensembles. Statistical tests were applied to the Kappa Temporal on both synthetic and real-world datasets, and significant differences were shown. Statistical test for Kappa M was also applied to both synthetic and real-world datasets and demonstrated significant differences with default values. The Nemenyi test [22] was applied for both Kappa Temporal and Kappa M for pairwise comparison. HDES-AD performed significantly better than the homogeneous ensemble approaches of AFWE, DDD, and OAUE. Even though DDD is an active homogeneous ensemble, it performs poorly in most of the datasets as a result of a weak drift detection mechanism that is not augmented by ensemble diversity. Apart from this, there was no significant difference between AFWE and OAUE. This makes HDES-AD independent of any base learner for classification problems in nonstationary time series data.

Tables 2 and 3 provide the Kappa measures for the experiments conducted.

The Kappa evaluation measure is widely used in evolving data streams as it can handle both multiclass and imbalanced class problems. The two tables indicate high values of the Kappa, a sign of a more generalized classifier. No negative values of the Kappa were recorded, and the prediction accuracy was on the higher side as the number of attributes in the datasets was of a reasonable number.

Table 3 shows the values of the Kappa M. The statistical tests applied to Kappa Temporal on artificial and real-world datasets showed significant differences. Statistical tests for Kappa M on both artificial and real-world datasets indicate significant differences. The Nemenyi test [22] was applied for both Kappa Temporal and Kappa M for pairwise comparison. The critical difference indicates that HDES-AD performs significantly better than the three homogeneous algorithms. Even though DDD is an active homogeneous ensemble, it performs poorly for predictable drifts and recurring concepts. Apart from this, there was no significant difference between OAUE and AFWE, OAUE, and HDES-AD.

The ensemble size in the HDES-AD algorithm is dynamic; that is, the ensemble is growing and shrinking based on the predictive performance and drift handling detection and capabilities. HDES-AD achieved higher accuracy on both synthetic and real-world datasets, and this can only be attributed to its heterogeneity. HDES-AD retains highly diverse classifiers, thus preserving previously learned concepts, and this helps HDES-AD to deal appropriately with recurring concept drifts. HDES-AD periodically includes new classifiers from the latest data chunks that are representative of the current concept to appropriately deal with concept drift and to maintain and improve predictive accuracy. DDD maintains a static ensemble and removes classifiers if its ensemble size reaches a predefined size, making it unable to handle recurrent and predictable drifts.

### 4.5. Accuracy over Time Plots

**Figure 2:**
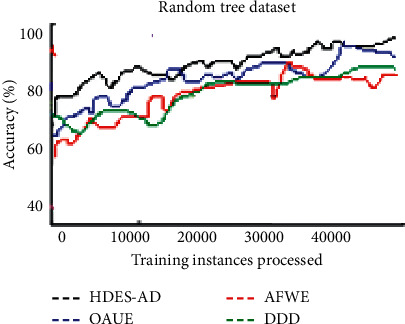
Predictive accuracy of the four algorithms on the SEA artificial data stream.

As shown in Figure 2, ADES achieved the highest predictive accuracy on SpamAssassin, KDD99, and Poker Hand. The average ranking of ADES-AD in real-world datasets is 1.5, OAUE is 3.25, AFWE is 3.25, and DDD is 3.0.

The accuracy over time plots of artificial data streams is shown in the figures that follow.

**Figure 3:**
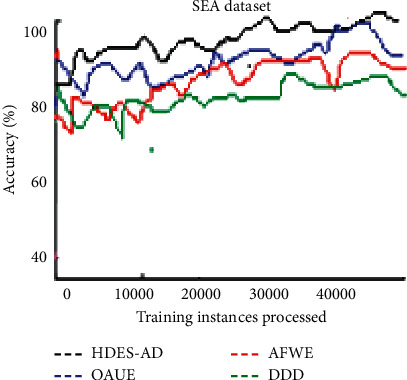
Predictive accuracy of the four algorithms on RandomTree artificial data stream.

Figure 3 shows the accuracy over time plot of the four algorithms on the Random Tree dataset which is devised to evaluate the algorithm's ability to adapt to recurring concepts. The prediction performance trend of all the algorithms is not different. Among them, HDES-AD adapts well to concept drift, followed by OUAE. AFWE performs the worst. HDES-AD adapts well to recurring concepts as it stores previously learned concepts for future use.

Figure 2 shows the accuracy over time plot of the four algorithms on the SEA dataset which is associated with sudden concept drifts. Although all algorithms experienced instantaneous fluctuations, OAUE performed well after the first 40000 observations were processed, followed by HDES-AD. The prediction performance of DDD and AFWE is almost identical although DDD is slightly less accurate.

**Figure 4:**
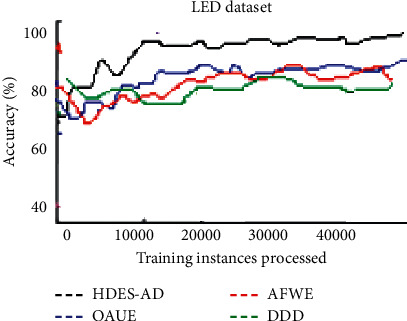
Predictive accuracy of the four algorithms on the LED artificial data streams.

Figure 4 demonstrates the accuracy of the four algorithms on the LED dataset which exhibits sudden concept drift. As can be observed, HDES-AD is the best, followed by OAUE. As the number of processed instances increases, DDD performs better than AFWE.

**Figure 5:**
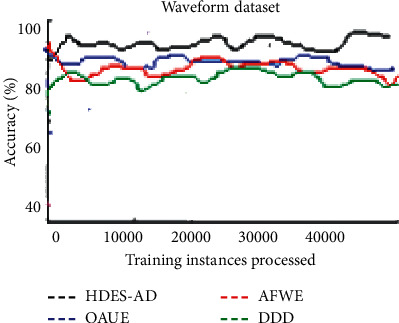
Predictive accuracy of the four algorithms on the waveform artificial data stream.

Figure 5 demonstrates the accuracy of the four algorithms on the Waveform dataset which is devised to evaluate the ability to adapt to sudden drifts. HDES-AD adapts to sudden drifts well as compared to the other three algorithms. The performance of the other three in adapting to sudden drifts is almost identical although AFWE is less accurate. The accuracy rates of all four algorithms fluctuate and suffer accuracy drops.

**Figure 6:**
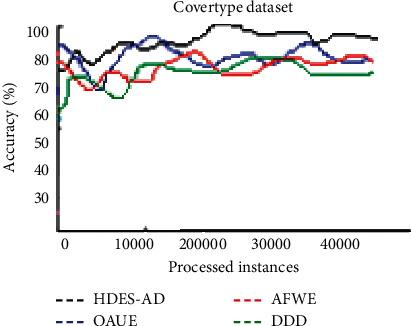
Average predictive accuracy on Covertype dataset.

The accuracy over time plots of the four algorithms on real-world datasets is shown in the figures that follow.  Figure 6 shows the accuracy over time plot of the four algorithms on the Covertype dataset. As more instances are observed, AFWE generalizes well to unseen instances. Despite fluctuations experienced by all algorithms, HDES-AD performs better than the other three algorithms. AFWE improves the accuracy as more instances are observed. DDD has the least performance in terms of accuracy.

**Figure 7:**
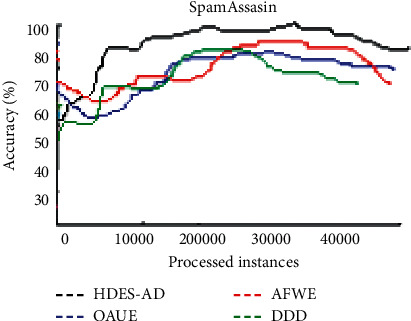
Average predictive accuracies on SpamAssassin dataset.

Figure 7 shows the accuracy over time plot of the four algorithms on the SpamAssassin dataset. After the observation of more than 40000 instances, the accuracy of all the four algorithms drops but HDES-AD maintains a higher stable accuracy. AFWE is second after HDES-AD. After more instances are observed, DDD accuracy fluctuates significantly.

**Figure 8:**
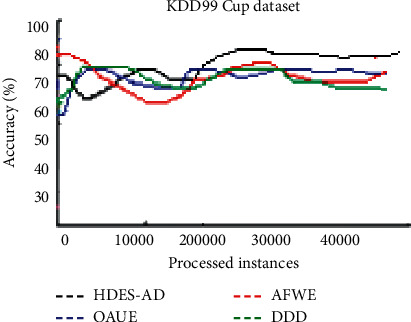
Average predictive accuracies on KDD99 dataset.

Figure 8 demonstrates the accuracy of the four algorithms on the KDD99 dataset. DDD performs significantly better in the first observations and the accuracy drops as more instances are observed. HDES-AD performs the best as observations increase. AFWE has the worst performance.

**Figure 9:**
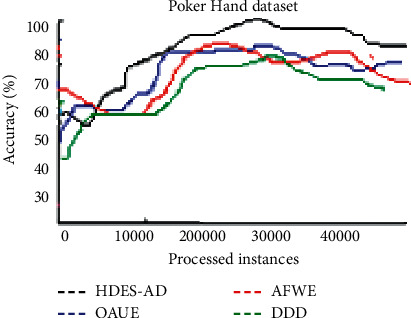
Average predictive accuracies on Poker Hand dataset.

Figure 9 shows the prediction performance of the four algorithms on the Poker Hand dataset. AFWE performs well when the first batch of observations is processed. HDES-AD performs the best followed by AFWE. DDD and OAUE performances are almost identical.

## 5. Analysis of Heterogeneity and Significance Difference

In this second experiment, the objective is to investigate the relationship between the heterogeneity of an ensemble and its predictive performance. We further analyze whether the higher prediction accuracy achieved in HDES-AD is a result of heterogeneity or is attributed to its active drift handling capabilities.

In this experiment, HDES-ADP is a variant of HDES-AD without active drift handling capabilities and relies on a passive approach similar to OAUE. The Friedman statistics using a heterogeneity test indicate significant differences. We applied the post hoc test using the Nemenyi test [22] for pairwise comparison. The critical difference (CD) shows that HDES-ADP performed significantly better than OAUE-MLP and OAUE-SVM.  Table 4 shows the heterogeneity test and prediction accuracy of the three algorithms.

**Figure 10:**
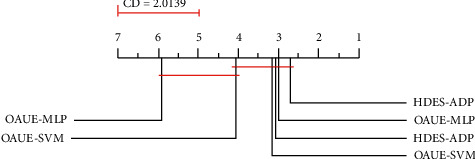
Average rank diagram for the compared approaches.

In Figure 10, we show the average rank diagrams of the compared approaches.

The algorithms that do not differ significantly are connected with a line. The critical difference (CD) is shown above the graph (CD = 2.0139).

The difference in prediction performance indicates that the main difference between HDES-ADP and OAUE is heterogeneity. These results provide an indication that heterogeneity plays a key role in improving the HDES-AD accuracy over OAUE. The availability of the model switching mechanism within the algorithm helps to maintain accuracy as the base classifiers are not selected manually.

## 6. Conclusion

The development of the Heterogeneous Dynamic Ensemble Selection based on Accuracy and Diversity (HDES-AD) for nonstationary time series data has opened new avenues of research in the area of handling concept drift. A heterogeneous online learning ensemble for nonstationary time series data called HDES-AD was designed to handle different types of concept drift using both passive and active approaches. The drift detection method (DDM) was used as a drift detection mechanism to test the drift handling capabilities. Homogeneous ensemble approaches used for comparative purposes were based on one learning algorithm and were selected using both passive and active approaches. The results show that, for some datasets, the HDES-AD algorithm is able to produce better predictive accuracy. The generalization performance of one ensemble approach over another seems to be highly problem-dependent. However, homogeneous ensemble approaches take time to reflect new concepts and require a large number of iterations to converge to new concepts. The difference that exists in terms of the accuracy of the predictions of the two ensemble approaches is negligible. HDES-AD maintains heterogeneous ensembles and is able to handle concept drifts due to its ability to create new learners and delete learners whose accuracy and diversity levels are below a predefined threshold. Although one ensemble approach performs better than the other in some real-world applications, there is no much significance in the difference in the performance of the ensemble algorithms under investigation.

## Figures and Tables

**Algorithm 1:**
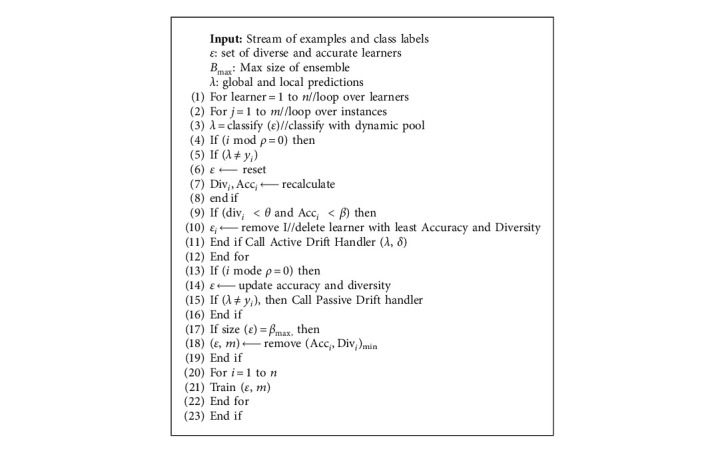
HDES-AD algorithm.

**Algorithm 2:**
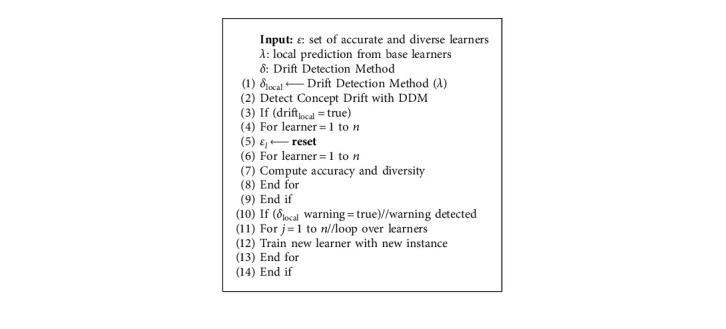
HDES-AD active drift handling.

**Algorithm 3:**
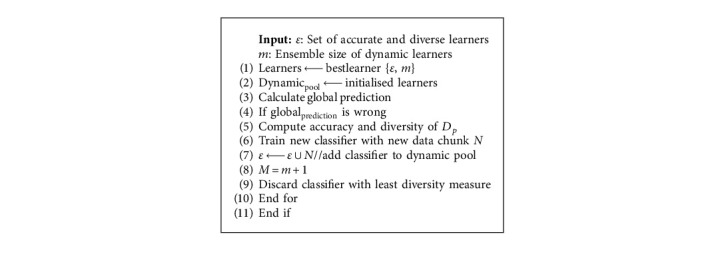
Passive handle drift.

**Table 1:** Predictive accuracies (%) of HDES-AD, DDD, OAUE, and AFWE.

Dataset	HDES-AD		DDD		OAUE		AFWE	
	Acc	CPU	Acc	CPU	Acc	CPU	Accuracy	CPU
Random	89.12 (1)	101.2	81.27 (2)	106.2	78.69 (4)	104.6	80.33 (3)	103.4
SEA	83.23 (1)	89.3	74.35 (3)	103.4	80.43 (2)	93.5	73.89 (4)	106.7
LED	81.34 (2)	134.6	76.54 (4)	159.6	82.34 (1)	148.3	76.67 (3)	138.4
Waveform	84.47 (1)	119.3	72.87 (3)	126.2	73.27 (3)	134.2	79.65 (2)	118.2
Covertype	85.59 (1)	108.3	78.43 (4)	114.6	82.35 (2)	113.4	71.23 (4)	139.3
SpamAssassin	78.93 (1)	120.2	73.17 (3)	128.3	69.74 (4)	142.7	74.43 (2)	134.4
KDD99	80.37 (1)	112.4	73.89 (4)	125.3	74.38 (3)	122.3	76.34 (2)	138.2
Poker Hand	76.87 (2)	104.6	78.67 (1)	115.2	7325 (3)	116.9	71.26 (4)	139.4
**Average ranks**	**1.25**		**3.0**		**2.75**		**3.0**	

**Table 2:** Kappa temporal evaluation measures on both artificial and real-world datasets.

Stream	HDES-AD	DDD	OAUE	AFWE
Random tree	76.48 (1)	71.34 (4)	74.23 (2)	72.43 (3)
SEA	81.37 (1)	74.38 (4)	76.43 (3)	78.35 (2)
LED	76.47 (1)	71.45 (2)	71.23 (4)	73.38 (3)
Waveform	68.34 (2)	69.42 (3)	70.48 (1)	66.48 (4)
Covertype	66.47 (3)	62.39 (4)	64.48 (3)	69.37 (2)
SpamAssassin	87.36 (1)	69.43 (3)	80.39 (2)	78.46 (4)
KDD99 Cup	83.42 (1)	72.48 (2)	69.23 (4)	71.13 (3)
Poker Hand	89.47 (1)	76.36 (3)	71.42 (2)	69.38 (4)
**Average ranks**	**1.75**	**3.13**	**2.63**	**3.12**

**Table 3:** Kappa evaluation measures on both artificial and real-world datasets.

Stream	HDES-AD	DDD	OAUE	HEFT
Random Tree	68.43 (1)	62.33 (4)	65.43 (2)	62.38 (3)
SEA	48.37 (1)	37.53 (4)	44.37 (3)	46.64 (2)
LED	66.48 (1)	64.38 (2)	62.48 (3)	59.38 (4)
Waveform	76.64 (1)	68.33 (3)	66.37 (4)	69.43 (2)
Covertype	64.38 (1)	59.36 (4)	63.46 (2)	61.34 (3)
SpamAssassin	84.46 (1)	78.37 (2)	74.56 (4)	76.37 (3)
KDD99 Cup	66.47 (3)	62.39 (4)	73.37 (1)	71.63 (2)
Poker Hand	64.45 (1)	47.64 (3)	49.43 (2)	47.83 (4)
**Average ranks**	**1.25**	**3.25**	**2.63**	**2.88**

**Table 4:** Heterogeneity test and predictive accuracy (%).

	HDES-ADP	OAUE-MLP	OAUE-SVM
Random Tree	84.37 (2)	86.78 (1)	80.43 (3)
SEA	81.34 (1)	77.68 (2)	74.58 (3)
LED	76.84 (1)	71.67 (3)	74.53 (2)
Waveform	74.48 (1)	74.44 (2)	74.41 (3)
Covertype	87.64 (2)	89.43 (1)	85.63 (3)
SpamAssassin	92.48 (1)	83.42 (3)	87.68 (2)
KDD99 Cup	86.78 (1)	82.46 (2)	79.89 (3)
Poker Hand	90.68 (1)	88.49 (3)	85.87 (2)
**Average ranks**	**1.25**	**2.13**	**2.63**

## Data Availability

The research used four artificial datasets, namely, Random Tree Generator, SEA Generator, LED Generator, and Waveform Generator. The real-world datasets used are Covertype dataset, Spam e-mail dataset, KDD99 Cup dataset, Poker Hand dataset. The artificial and real-world data used to support the findings of this study have been deposited in the following repositories and sources: (1) Random Tree Generator—Cunningham P., Nowlan N., Delany S. J., and Haahr M., 2003. A case-based approach to spam filtering that can track concept drift. In the Proceedings of ICCBR-2003 Workshop on Long-Lived CBR Systems.; https://www.cs.waikato.ac.nz/∼abifet/MOA/API/classmoa_1_1streams_1_1generators_1_1_random_tree_generator.html; (2) SEA Generator—https://moa.cms.waikato.ac.nz/details/classification/streams/; Wang H., Fan W., Yu P. S., and Han J., 2003. Mining concept-drifting data streams using ensemble classifiers, In proceedings of 9^th^ ACM SIGKDD Int. Conf. on Knowledge Discovery and Data Mining KDD-2003, ACM Press, pp: 226–235; (3) LED Generator—Cunningham P., Nowlan N., Delany S. J., and Haahr M., 2003. A case-based approach to spam filtering that can track concept drift. In the Proceedings of ICCBR-2003 Workshop on Long-Lived CBR Systems; (4) Waveform Generator—Cunningham P., Nowlan N., Delany S. J., and Haahr M.,2003. A case-based approach to spam filtering that can track concept drift. In the Proceedings of ICCBR-2003 Workshop on Long-Lived CBR Systems: Covertype dataset is available at https://archive.ics.uci.edu/ml/datasets/Covertype; Spam e-mail dataset at http://spamassassin.apache.org/;KDD99 dataset at http://kdd.ics.uci.edu; and Poker Hand dataset at https://archive.ics.uci.edu/ml/datasets/Poker+Hand.
